# Evidence supporting a role for astrocytes in the regulation of cognitive flexibility and neuronal oscillations through the Ca^2+^ binding protein S100β

**DOI:** 10.1371/journal.pone.0195726

**Published:** 2018-04-17

**Authors:** Adam T. Brockett, Gary A. Kane, Patrick K. Monari, Brandy A. Briones, Pierre-Antoine Vigneron, Gabriela A. Barber, Andres Bermudez, Uma Dieffenbach, Alexander D. Kloth, Timothy J. Buschman, Elizabeth Gould

**Affiliations:** 1 Princeton Neuroscience Institute and Department of Psychology, Princeton University, Princeton, NJ, United States of America; 2 Department of Cell Biology and Physiology and Neuroscience Center University of North Carolina at Chapel Hill, Chapel Hill, NC, United States of America; University of Louisville, UNITED STATES

## Abstract

The medial prefrontal cortex (mPFC) is important for cognitive flexibility, the ability to switch between two task-relevant dimensions. Changes in neuronal oscillations and alterations in the coupling across frequency ranges have been correlated with attention and cognitive flexibility. Here we show that astrocytes in the mPFC of adult male Sprague Dawley rats, participate in cognitive flexibility through the astrocyte-specific Ca^2+^ binding protein S100β, which improves cognitive flexibility and increases phase amplitude coupling between theta and gamma oscillations. We further show that reduction of astrocyte number in the mPFC impairs cognitive flexibility and diminishes delta, alpha and gamma power. Conversely, chemogenetic activation of astrocytic intracellular Ca^2+^ signaling in the mPFC enhances cognitive flexibility, while inactivation of endogenous S100β among chemogenetically activated astrocytes in the mPFC prevents this improvement. Collectively, our work suggests that astrocytes make important contributions to cognitive flexibility and that they do so by releasing a Ca^2+^ binding protein which in turn enhances coordinated neuronal oscillations.

## Introduction

Cognitive flexibility, the ability to shift between two task relevant response sets, is dependent on the mPFC and is impaired in patients with mood disorders [1] and schizophrenia [2]. Patients with mood disorders and schizophrenia often present with abnormal gamma (30–80 Hz) and theta (6–10 Hz) oscillations which are thought to support cognitive flexibility and mPFC function [3]. In the mPFC, decreases in astrocyte number have been reported in patients with mood disorders [4,5], and schizophrenia [6,7], and the magnitude of these changes often exceeds those reported for neurons [8].

Despite their historical characterization as support cells, astrocytes are complex and highly heterogeneous cells capable of regulating synaptic strength [9,10]. Astrocytes release molecules known to play an important role in long term potentiation (LTP) [11–13] and have been associated with the regulation of neuronal oscillations and rhythmic firing [14–17]. These findings demonstrate that astrocytes are active participants in brain function, but the importance of astrocytes in complex behaviors has been less explored.

Previous studies have shown that reducing astrocyte numbers in the mPFC of rodents using a specific astrocytic toxin, _L_-alpha aminoadipic acid (L-AAA), leads to anhedonia and altered cognitive ability [18,19], while chemogenetic enhancement of astrocytic Ca^2+^ signaling in the nucleus accumbens reduces addiction-like behaviors [20,21]. Collectively, these findings suggest that astrocyte number and physiology may be important for complex behavior, including cognition. However, whether astrocytes in the mPFC support cognitive flexibility through the regulation of neuronal oscillations is unclear. Here we sought to investigate this question directly. Using the astrocyte specific toxin, L-AAA, we found that a reduction in astrocyte number in the mPFC was associated with impaired cognitive flexibility and reduced power across delta (1–4 Hz), alpha (12–20 Hz), and gamma (30–80 Hz) frequency ranges. Next, we explored whether activation of astrocytic Ca^2+^ signaling using designer receptors exclusively activated by designer drugs (DREADD), in the mPFC was sufficient to improve cognitive flexibility, and found supportive evidence. The astrocyte-specific Ca^2+^ binding protein S100β has been implicated in the regulation of rhythmic firing in neurons [16], which we hypothesized may be important for supporting cognitive flexibility. We found that blocking the activity of S100β after chemogenetic enhancement of astrocytic Ca^2+^ signaling prevented the beneficial effects of DREADD activation on cognitive flexibility. In line with these findings, we further showed that infusion of S100β into the mPFC improved cognitive flexibility, and was associated with increased coupling between theta and gamma oscillations. Collectively, these results provide evidence linking an astrocyte-specific molecule to both behavior and neuronal signaling and suggest that astrocytes in the mPFC play an important role in cognitive function.

## Materials and methods

### Animals

Adult male (7–9 week old) Sprague-Dawley rats were used for these studies. Rats were purchased from Taconic Biosciences (Rensselaer, NY) and bred for research purposes. Rats were housed individually after surgery on a reverse 12-hour light-dark schedule (lights off at 0700), and all behavioral testing and recordings occurred between 0900 and 1400. All studies were approved by the Princeton University IACUC and conformed to the National Research Council Guide for the Care and Use of Laboratory Animals (2011).

### Behavioral testing

All rats completed the attentional set-shifting task (ASST), a well-characterized task of cognitive flexibility, the details of which have been described previously [22,23]. Briefly, the ASST consists of 3 days of testing in which rats learn to discriminate between two different exemplars in order to retrieve a food reward (1/4 of a Froot Loop). Before each day of testing, rats were habituated to the testing room for 10 minutes. The ASST consists of 5 separate discriminations: simple discrimination, compound discrimination, intradimensional shift, reversal, and extradimensional shift. For each phase, rats must reach a criterion of 6 consecutive trials of correctly retrieving the food reward before advancing to the next phase. Critically, performance on the extradimensional shift is thought to most closely resemble cognitive flexibility and is dependent on the mPFC [22,23]. For all experiments in which rats completed the ASST, astrocytes in the mPFC were manipulated and performance on the ASST was examined. All rats, regardless of treatment, were able to successfully complete all of testing by the end of the third day.

### Astrocyte manipulations

For all ASST behavioral experiments rats were anesthetized with isoflurane, shaved and placed in a stereotaxic instrument (David Kopf Instruments). Holes were drilled into the skull using the following coordinates: 2.7 mm anterior to bregma, ±0.5 mm lateral to the midline, and 4.0 mm ventral from the dura [18]. In separate cohorts of rats, astrocytes in the mPFC were manipulated using one of the following strategies, before either being tested on the ASST or undergoing neural recordings.

### Lesion of astrocytes in the mPFC

L-AAA is an astrocyte-specific toxin that can reduce astrocyte numbers for up to two months [18,24]. L-AAA (Sigma) was dissolved in sterile 1M HCl at a concentration of 20 μg/μl [24]. The solution was sonicated at 30 Hz for 10 minutes before being titrated with sterile 2M NaOH (pH = 7.5). Rats received either 1 μl of L-AAA (n = 12) or saline (n = 11) infused bilaterally over the course of five minutes into the mPFC. One hour following the completion of behavioral testing, rats were deeply anesthetized and perfused with 4% paraformaldehyde and their brains were processed for histology and microscopic analysis.

In a separate control experiment, a new cohort of rats (n = 6) received 1 μl injections of L-AAA (20 μg/μl) or saline in the mPFC and were allowed 13 days to recover before being perfused with 1.5% paraformaldehyde for diolistic analysis of spine density. This control was designed to verify whether L-AAA had unintentional negative consequences on dendritic plasticity.

### DREADD activation of astrocytes in the mPFC

Rats (n = 15) received 1 μl bilateral infusions of rAAV5/GFAP-HA-hm3D-IRES-mCitrine (GFAP-Gq-DREADD) (University of North Carolina Vector Core, Chapel Hill, North Carolina) into the mPFC at a concentration of 4.0 x 10^12^ particles/ml [20,21]. 20 minutes prior to completing the extradimensional shift, (4 weeks post-surgery), rats received either a 3 mg/kg IP injection of clozapine-N-oxide (CNO) (n = 8), a concentration known to maximally activate the GFAP-Gq-DREADD construct [20,21] or saline (n = 7). Rats were returned their home cages and given a five-minute recovery period before completing testing.

To rule out the possibility that CNO, independent of GFAP-Gq-DREADD activation, was contributing to changes in cognitive flexibility a new cohort of non-infected control rats was run on the ASST. 20 minutes prior to completion of the extradimensional shift, animals received an IP injection of either CNO (3 mg/kg) (n = 6) or a comparable volume of saline (n = 6). Five minutes following the injection of either CNO or saline rats completed the rest of the ASST before being perfused one day later.

### Manipulation of S100β in the mPFC

S100β, is an astrocyte specific protein and has an established role in the regulation of intracellular, and more recently, extracellular Ca^2+^ [16]. S100β (Sigma, S6677) or a physiologically inert form of S100β that is incapable of binding Ca^2+^, mS100β (a generous gift from D. Weber from the Center for Biomolecular Therapeutics, University of Maryland School of Medicine), was dissolved in sterile saline at a concentration of 1 mM [16,25]. Rats were fit with cannulae bilaterally targeting the mPFC. Three self-tapping bones screws (FST) were placed surrounding the implant. A base layer of MetaBond (Parkell) was applied to the skull and implant and allowed to dry before dental acrylic (Bosworth) was used to build up the skull cap. Rats were food-deprived to 85% body weight, and were infused with either S100β (n = 12) or mS100β (n = 12) into the mPFC 20 minutes prior to testing on the extradimensional shift. Rats were briefly restrained and received a 1 μl infusion of S100β bilaterally over the course of one minute. Flow rate was controlled by a syringe pump driver (Harvard Apparatus). Following infusion, the internal cannulae were left in place for five minutes before being removed in order to minimize backflow. Dummy caps were reattached and rats were given a five-minute break before continuing testing.

Rabbit x S100β (Abcam, ab52642) or non-immune rabbit IGG (Sigma, I5006) was diluted to a concentration of 40 μg/ml [16]. Rats received 1 μl bilateral infusions of GFAP-Gq-DREADD into the mPFC at a concentration of 4.0 x 10^12^ particles/ml, and were then fit with cannulae bilaterally targeting the mPFC. Testing began three weeks after surgery in order to ensure adequate time for GFAP-Gq-DREADD expression. Rats were food-deprived to 85% body weight over five days before the start of behavioral testing. Behavioral testing occurred over the course of three days (habituation, shaping, and testing). On the testing day, 20 minutes prior to the extradimensional shift (4 weeks post-surgery), rats received a 3 mg/kg IP injection of clozapine-N-oxide (CNO), a concentration known to maximally activate the GFAP-Gq-DREADD construct [20,21]. Immediately following the injection of CNO, rats were lightly restrained and received a 1 μl infusion of either antibody against S100β (n = 9) or non-immune rabbit IGG (Sigma) (n = 9) into the mPFC. Following infusion, the internal cannulae were left in place for five minutes before being removed in order to minimize backflow. Dummy caps were reattached and rats were given a 10-minute break before continuing testing.

### LFP recordings and analysis

In a separate set of experiments, we recorded local field potentials (LFPs) from astrocyte manipulated rats that had no previous experience with behavioral testing. Based on our previous behavioral findings, we chose to manipulate astrocytes in one of two ways; either by reducing the number of astrocytes using L-AAA or by infusing S100β. The version of the ASST that we used for our behavioral experiments is not amenable to recording experiments given the requirement that animals must dig in a food bowl in order to receive reward. Therefore, we chose to record LFPs from rats exploring a behavior box (17 x 17 x 17 cm). Rats were habituated to the recording cable, and all recordings occurred in the dark under red light so as not to minimize anxiety-provoking stimuli.

In experiment one, rats received bilateral infusions of L-AAA (20 μg/ μl) (n = 8) or saline (n = 8) and were fit a single 125 μm insulated stainless-steel electrode (Plastics One), unilaterally targeting mPFC. Hemispheres were counterbalanced and LFPs were recorded for 20 minutes while rats roamed the behavior box. In experiment two, rats (n = 18) were implanted with a dual cannula electrode system (Plastics One) unilaterally targeting mPFC. Implanted hemispheres were counterbalanced. On recording days, rats received either an infusion of S100β or mS100β directly into mPFC. 20 minutes after infusion, rats were placed in the behavior box for 20 minutes, LFPs were recorded, then rats were returned to their home cage. Treatment order was counterbalanced.

LFP recording data were collected from the three separate recording sessions and analyzed using a between subject design (L-AAA Recordings) or a within subject design (S100β Recordings). LFPs were recorded against a ground screw placed above the hippocampus. Signals were passed through a commutator, amplified 100X (CEW Inc), and sampled at 1000 Hz using a Cambridge Electronic Design 1401 data acquisition unit and Spike2 software. Signals were notch filtered at 60 Hz, and artifacts related to movement, detected using a voltage threshold, were cleaned from the data. Power spectral density estimates were obtained using short time Fourier transform. To obtain power estimates within delta, theta, alpha and gamma bands, we took the average power across time for the entire session within each frequency, then took the sum of this average power across the entire frequency band. To examine changes in individual bands power between treatments, paired t-test for were performed on the sum of power within individual frequency bands, respectively, for each animal.

For phase amplitude coupling analyses, theta (6–10 Hz) or alpha (12–20 Hz) and gamma (30–80 Hz) signals were filtered separately using a band-pass Butterworth filter (4^th^ Order), and analytic signals were obtained using the Hilbert transformation. Instantaneous theta or alpha phase was measured as the angle of the analytic theta signal, and instantaneous gamma amplitude was measured as the absolute value of the magnitude of the analytic gamma signal. To calculate the modulation index, we measured average gamma amplitude within theta or alpha frequency bins (each bin 3.6 degrees), and took the difference between the maximum gamma amplitude and minimum gamma amplitude as a function of theta or alpha frequency.

### Immunolabeling for glial and neuronal markers

In all experiments, rats were injected with an overdose of Euthasol and transcardially perfused with 4% paraformaldehyde, one hour after the completion of behavioral testing unless otherwise noted. Brains for immunohistochemical analysis were postfixed for 48 hours before processing. 40 μm coronal sections were stained using the following antibodies; astrocyte markers: rabbit anti-glial fibrillary acidic protein (GFAP) (1:500, Dako, z0334) or rabbit anti-S100 (1:10,000, Dako, zo311); neuronal marker: mouse anti-NeuN (1:500, Millipore, MAB377); immediate early gene markers: mouse anti-arc (1:500, SySy Systems, 156–003) or rabbit anti-egr-1 (1:500, Santa Cruz Biotechnology, sc-110), mouse anti-c-Fos (1:200, Santa Cruz Biotechnology, sc-166940); or microglial markers: rabbit anti-Iba1 (1:500, Wako, LKL0566), rat anti-CD68 (1:200, BioRad, MCA1957) and stored at 4°C. After incubation in primary antisera for 24 hours, sections were rinsed, incubated in either Alexa Fluor donkey anti-rabbit 488 (1:250; Invitrogen, A21206), Alexa Fluor donkey anti-rabbit 568 (1:250; Invitrogen, A10042), Alexa Fluor donkey anti-mouse 568 (1:250; Invitrogen, A10037) or Alexa Fluor donkey anti-rat 594 (1:250; Invitrogen, A21209) in the dark for 60 minutes, rinsed, mounted onto suprafrost slides, and coverslipped using glycerol in PBS (3:1). All sections were counterstained with the DNA dye, Hoechst 33342 (1:100,000, Molecular Probes, H1399). Slides were coded until completion of the data analysis. Images from the mPFC were taken using a Zeiss confocal microscope (LSM 700; lasers, argon 458/488; HeNe 568).

Measurement of astrocyte density, cross-sectional area, and immediate early gene expression were made using ImageJ (NIH). Density measurements of GFAP+, Arc+, Egr-1+, Iba1+, NeuN+ and S100+ cells were completed on representative sections throughout the mPFC. Density measurements were taken from cells surrounding the layer 2/3 pyramidal neurons and were obtained from 20 μm image stacks using ImageJ (NIH). Cross-sectional area measurements of 50 GFAP+, NeuN+ and S100+ cells per animal were obtained from 20 μm image stacks using ImageJ (NIH) [23]. Selected cells had to be distinct from surrounding labeled cells and be fully stained. Optical intensity measures for CD68+ were analyzed by collecting mean intensity values from 1 μm optical sections [23]. All parameters for background subtraction were optimized and held constant throughout the analysis.

### DiI labeling

In two separate control experiments, we assessed potential changes in dendritic spine density following either L-AAA treatment (n = 6), or due to CNO activation of the GFAP-Gq-DREADD (n = 10). Individual sections were shot with lipophilic DiI crystals (Sigma, 486495) using the Helios Gene Gun System (BioRad) as previously described [26]. Sections were visualized using a Zeiss confocal microscope (LSM 700; lasers, argon 458/488; HeNe 568) with a 63x oil objective. All settings (pinhole size, aperture gain, and offset) were initially optimized and held constant throughout the study.

### Experimental design and statistical analysis

Unpaired two tailed Student’s t-tests were performed on each data set (for cellular analyses: saline x astrocyte manipulated), except for spine type data and the ASST data. Spine type data were analyzed with Student t-tests (saline vs. CNO) and corrected for multiple comparisons using a Holm-Sidak correction. The ASST data were analyzed as a mixed factorial ANOVA with Holm-Sidak correction for multiple comparisons where appropriate. Student t-tests were used to assess differences in power and for phase-amplitude coupling analyses, differences in modulation index between treatments were tested using a two-sided Wilcoxon signed rank test.

## Results

### Reduction of astrocyte number is associated with impaired cognitive flexibility and diminished gamma power

**Fig 1:**
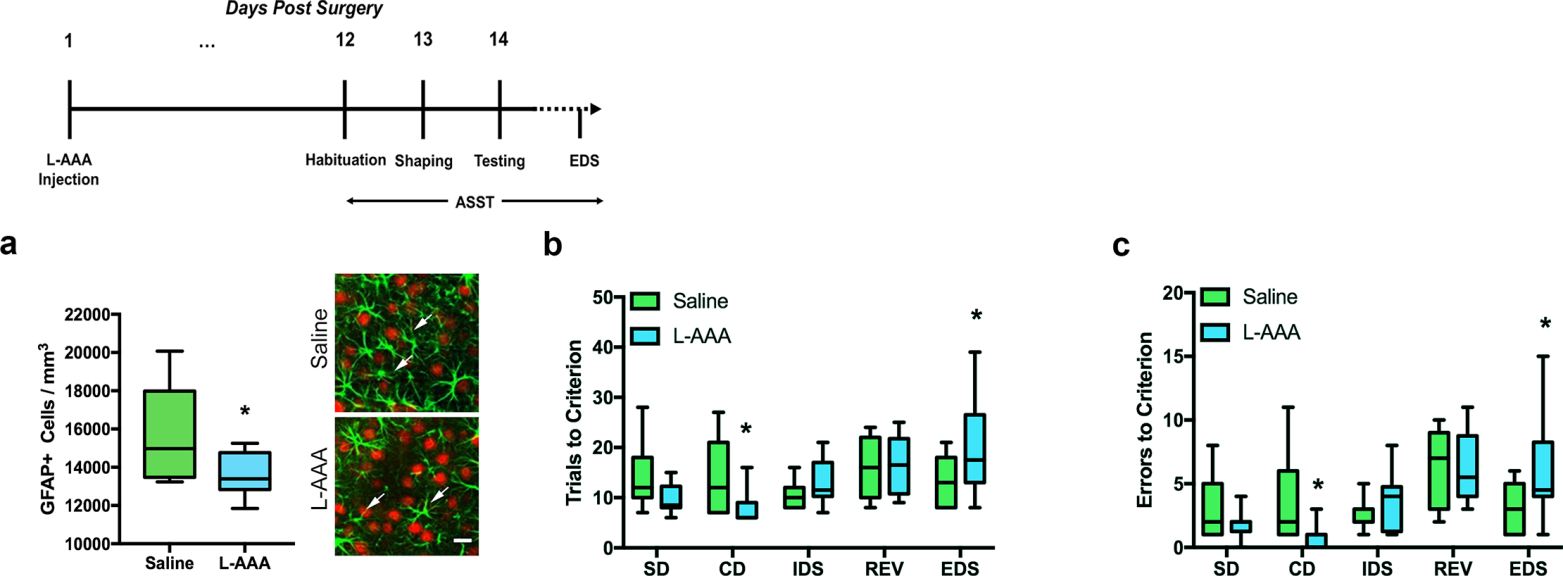
Reduction of astrocyte number in the mPFC impairs cognitive flexibility. Rats were infused with either L-AAA (n = 12) or saline (n = 11) in the mPFC 12 days before completing the ASST. L-AAA treatment altered mPFC-dependent performance on the extradimensional shift (EDS). A timeline for the L-AAA behavioral experiment is provided above. ***a*.** L-AAA treatment significantly reduced the number of GFAP+ astrocytes in the mPFC. Confocal images of GFAP+ (green) and NeuN+ (red) staining 13 days after treatment with either L-AAA or saline. Scale bar = 20 μm. * p<0.05 compared to saline-infused controls. Arrows point to either the presences or absence of GFAP+ staining in green. ***b*.** Rats with fewer astrocytes in the mPFC took fewer trials to reach criterion for the compound discrimination (CD) compared to saline-infused controls but more trials to reach criterion on the EDS portion of the task when compared to saline controls. ***c*.** Rats with fewer astrocytes also made fewer errors on the CD, but more errors on the EDS compared to saline-infused controls.

L-AAA significantly reduced the density of GFAP+ astrocytes in the mPFC (*t*_(21)_ = 2.771, *p* = .0115) (Fig 1A). Moreover, reduction of astrocyte number impaired performance on the extradimensional shift as evidenced by increases in trials to criterion (*p* = .0260) and errors made (*p* = .0276) (Fig 1B and 1C). Astrocyte reduction enhanced performance on the compound discrimination task by reducing both trials to criterion and errors made (trials to criterion: *p* = .0375, errors made: *p* = .0301) (Fig 1B and 1C). Taken together, these findings suggest that a small (~20%) reduction in the number of astrocytes in the mPFC hinders cognitive flexibility.

**Fig 2:**
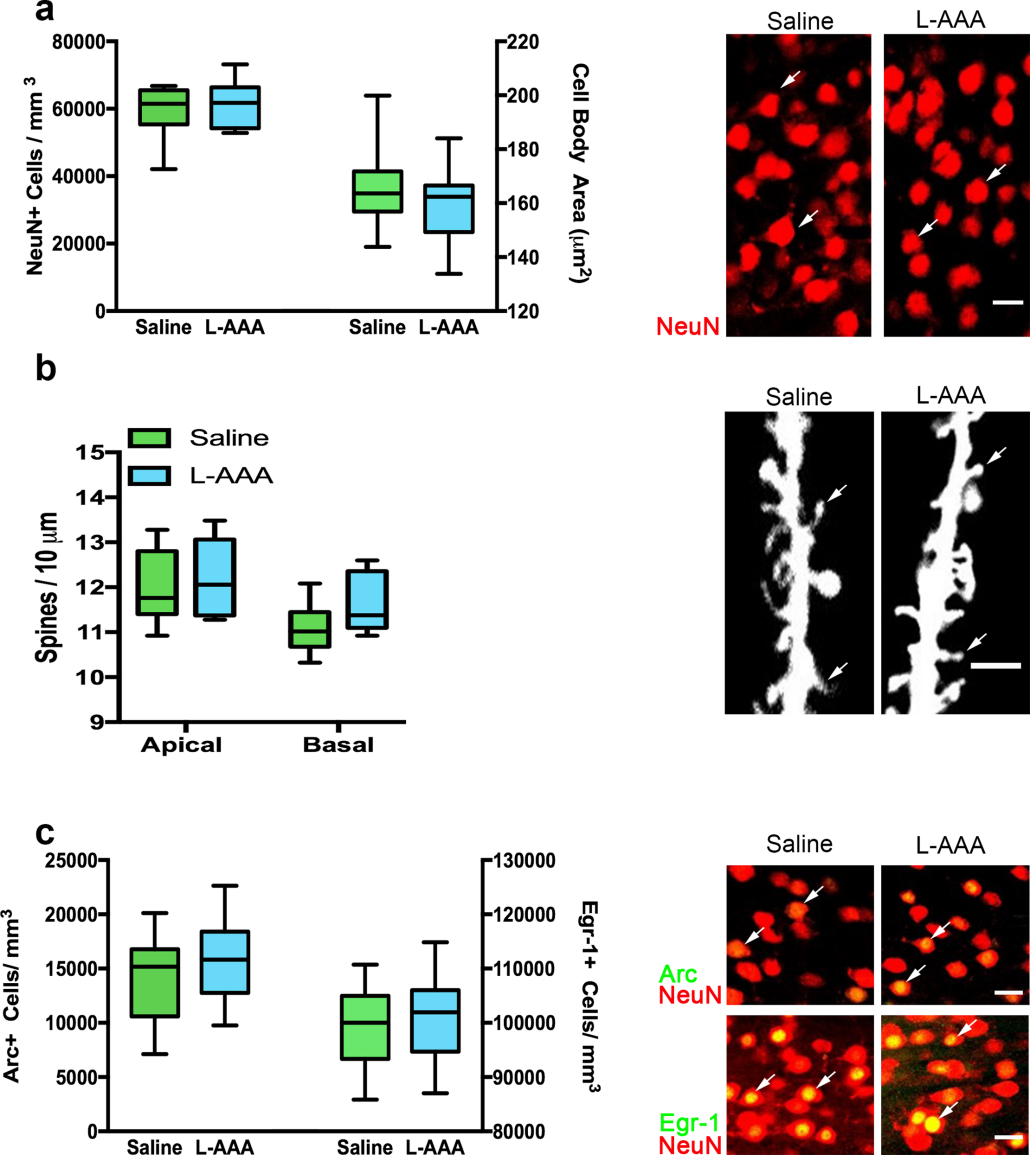
L-AAA does not alter neuron number, size, dendritic spine density or immediate early gene expression in the mPFC. To rule out the possibility that L-AAA had deleterious effects on neurons, neuronal viability was characterized using several measures. ***a*.** Relative to saline-infused controls, both the density and cell body size of NeuN+ layer 2/3 pyramidal neurons was unchanged in L-AAA treated rats (n = 11–12). Confocal images of NeuN+ staining in the mPFC. Scale bar = 20 μm. Arrows point to NeuN stained cell bodies in the mPFC. ***b*.** In a separate cohort of rats, no differences in dendritic spine density for both apical and basal secondary and tertiary dendrites of layer 2/3 pyramidal neurons were found when comparing saline and L-AAA treated rats (n = 6). Confocal images of DiI-labeled tertiary apical dendrites in layer 2/3 pyramidal neurons in the mPFC. Scale bar = 5 μm. Arrows point to example spines on along the dendrite. ***c*.** No differences in the density of Arc+ and Egr-1+ neurons in layer 2/3 of the mPFC were detected between saline and L-AAA -treated rats (n = 11–12) after completing the ASST. Confocal images of Arc (green) and Egr-1 (green) and NeuN (red) immunolabeling. Scale bar = 20 μm. Arrows point to examples of colabeling between NeuN+ neurons in red and the respective immediate early gene marker in green.

**Fig 3:**
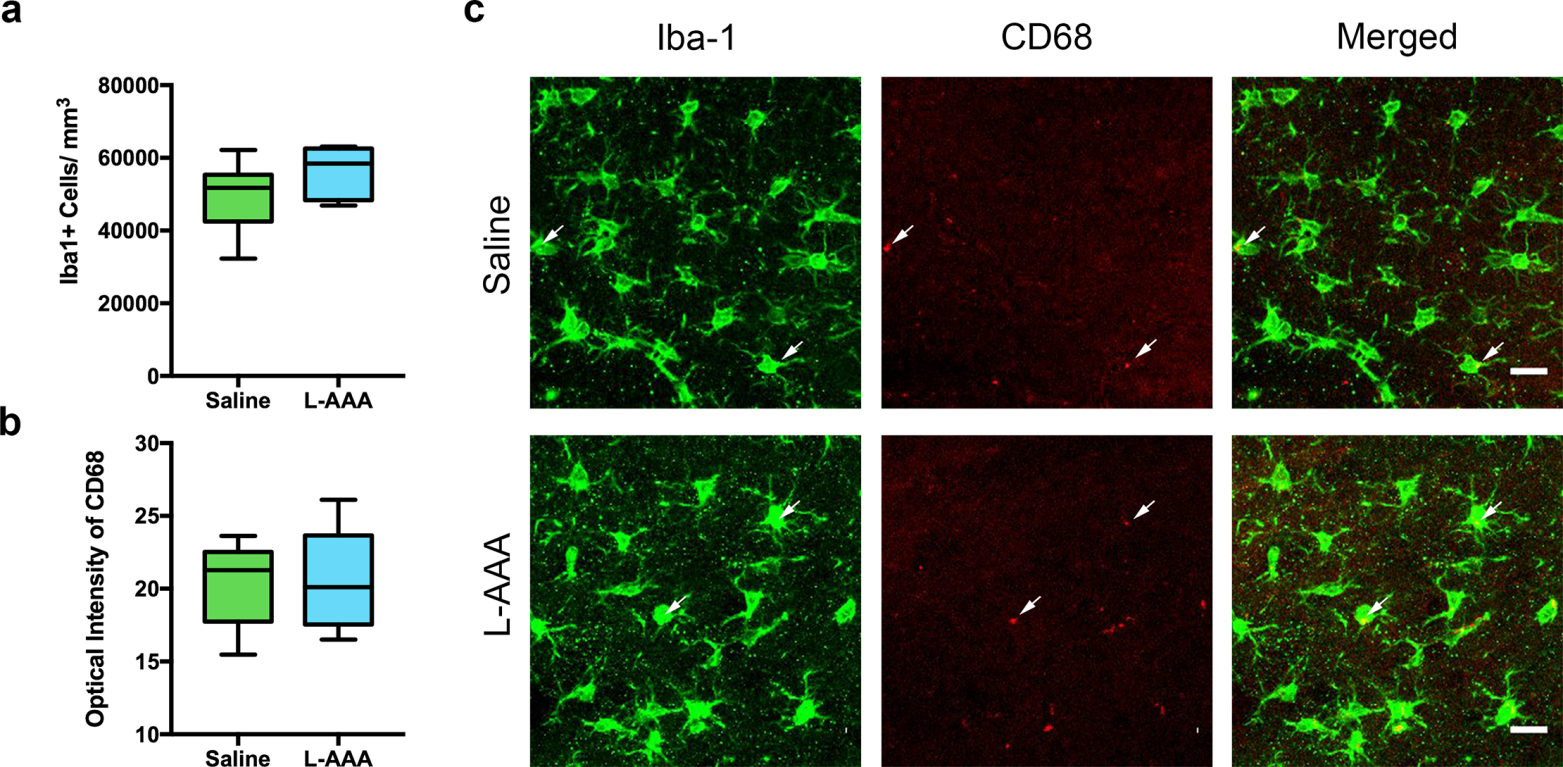
L-AAA does not alter microglial number or CD68 expression in the mPFC. To rule out the possibility that L-AAA had deleterious effects on microglia, microglial activation was characterized using several measures. ***a*.** Relative to saline-infused controls, the density of Iba1+ microglia was unchanged in L-AAA treated rats (n = 8). ***b*.** No differences in optical intensity measures of the microglial lysosomal marker CD68 were found when comparing saline and L-AAA treated rats (n = 8). ***c*.** Confocal images of Iba1 (green) and CD68 (red) immunolabeling in the mPFC of saline and L-AAA treated rats. Scale bar = 20 μm. Arrows point to example microglia labeled with Iba1 in green that colabel with vesicle marker CD68 in red.

L-AAA did not alter the number of mPFC NeuN+ neurons (*t*_(21)_ = .5333, *p* = .5994); the size of mPFC neuronal cell body areas (*t*_(21)_ = .9433, *p* = .3563), the densities of dendritic spines on apical (*t*_(10)_ = .4277, *p* = .6779) and basal (*t*_(10)_ = 1.473, *p* = .1715) dendrites of layer 2/3 mPFC pyramidal neurons, or the expression of the immediate early genes Arc and Egr-1, proxies for neuronal activation after ASST (Arc: *t*_(21)_ = .9752, *p* = .3405; Egr-1: *t*_(21)_ = .4786, *p* = .6372) in layer 2/3 mPFC pyramidal neurons (Fig 2A–2C). L-AAA treatment did not alter the density of Iba1+ microglia (*t*_(14)_ = 1.645, *p* = .1222), nor did it change the presence of CD68 (*t*_(14)_ = .1519, *p* = .8815), two indicators of microglial reactivity (Fig 3A–3C). Collectively, these findings suggest that both neurons and microglia in the mPFC were unchanged morphologically and that neurons were functional at the time of behavior.

**Fig 4:**
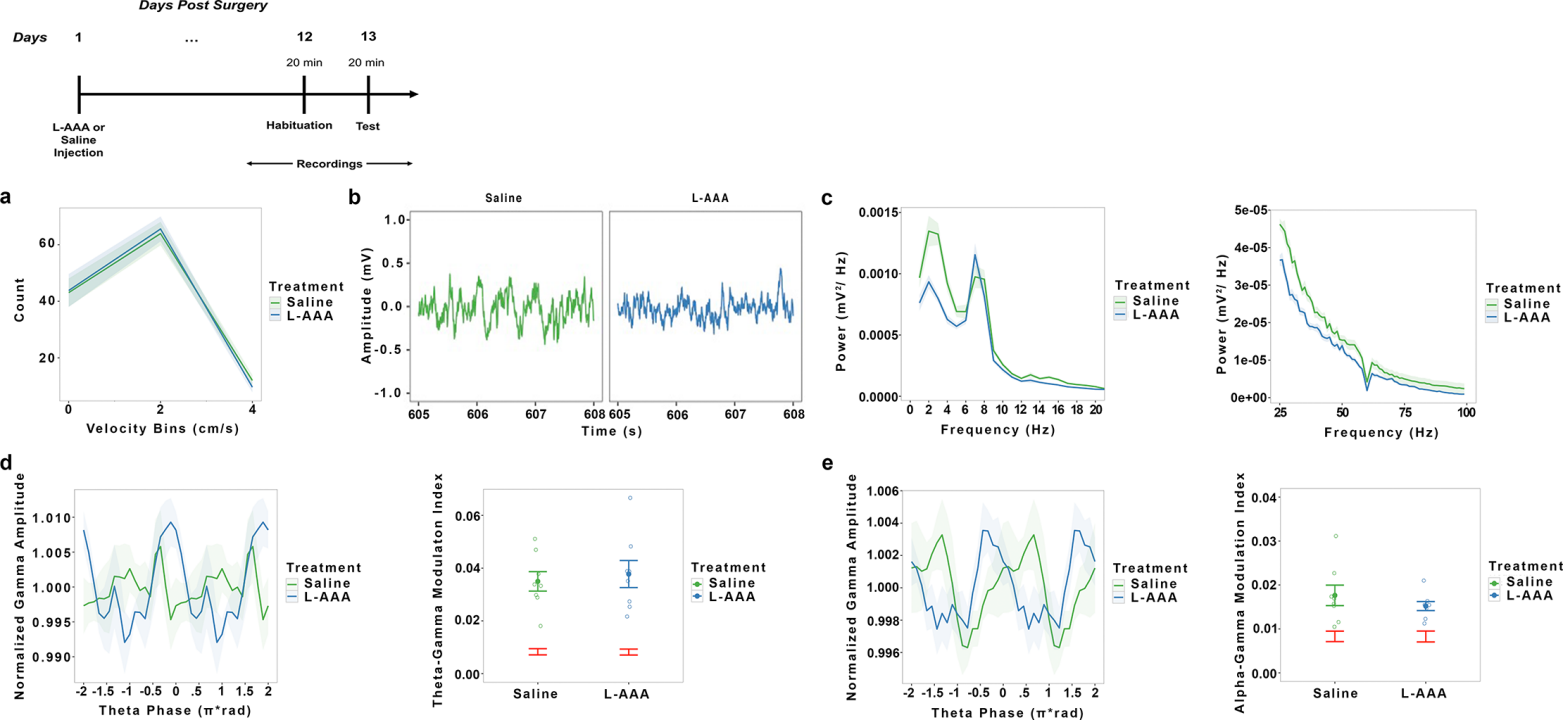
L-AAA reduces gamma power but does not affect phase-amplitude coupling between theta and gamma or alpha and gamma oscillations in the mPFC. To examine whether L-AAA altered the coupling between theta-gamma or alpha-gamma frequency ranges, we conducted two phase amplitude coupling analyses to examine the relationship between theta and gamma and alpha and gamma oscillations. LFPs were recorded while rats (n = 8) roamed an open field after L-AAA for saline treatment twelve days earlier. A timeline for the experiment is provided above. ***a*.** A mixed effects model revealed no differences in movement across velocity bins as a function of treatment. ***b*.** Raw LFP recordings from saline treated and L-AAA animals. ***c*.** L-AAA reduced power in the delta and alpha frequency ranges p<0.05. L-AAA reduced power in the gamma frequency range p<0.05. ***d*.** Phase amplitude coupling analysis plotting normalized gamma power as a function of theta phase. Permutation results showed significant modulation above chance for both treatment conditions (red). L-AAA did not significantly change theta-gamma modulation relative to saline controls suggesting that L-AAA effects on neuronal oscillations may be specific to only the power of individual frequency ranges and not the interaction amongst frequency ranges. ***e*.** Phase amplitude coupling analysis plotting normalized gamma power as a function of alpha phase. Permutation results showed significant modulation above chance for both treatment conditions (red), suggesting that L-AAA treatment did not alter synchrony between alpha and gamma oscillations.

Astrocytes have been shown to be robust regulators rhythmic firing patterns in neurons [14–17]. We hypothesized that a reduction in astrocyte number may alter the power or frequency of neuronal oscillations associated with task performance. We found no effect of treatment on locomotor activity (*t*_(14)_ = .9229, *p* = .3717) (Fig 4A). Reduction of astrocyte number in the mPFC diminished power in delta (*t*_(14)_ = 2.568, *p* = .0223), alpha (*t*_(14)_ = 4.339, *p* = .0007), and gamma (*t*_(14)_ = 2.484, *p* = .0263) frequency ranges, but not in theta (*t*_(14)_ = .9717, *p* = .3477) (Fig 4B and 4C). We also investigated whether synchrony between theta and gamma and alpha and gamma oscillations were altered by a reduction in astrocytes. Previous research suggests that improvement in memory and attentional abilities in the mPFC are correlated with enhanced synchrony between theta and gamma oscillations as well as the harmonic (alpha and gamma) oscillations [27,28]. We performed two phase amplitude coupling analyses, examining changes in synchrony between theta and gamma and alpha and gamma oscillations and found no differences in synchrony between L-AAA and saline treated controls (theta and gamma (*z*_(14)_ = -.2554, *p* = .7984); alpha and gamma oscillations (*z*_(14)_ = -.8737, *p* = .3823))(Fig 4D and 4E).

### DREADD activation of astrocytes in the mPFC improves cognitive flexibility

**Fig 5:**
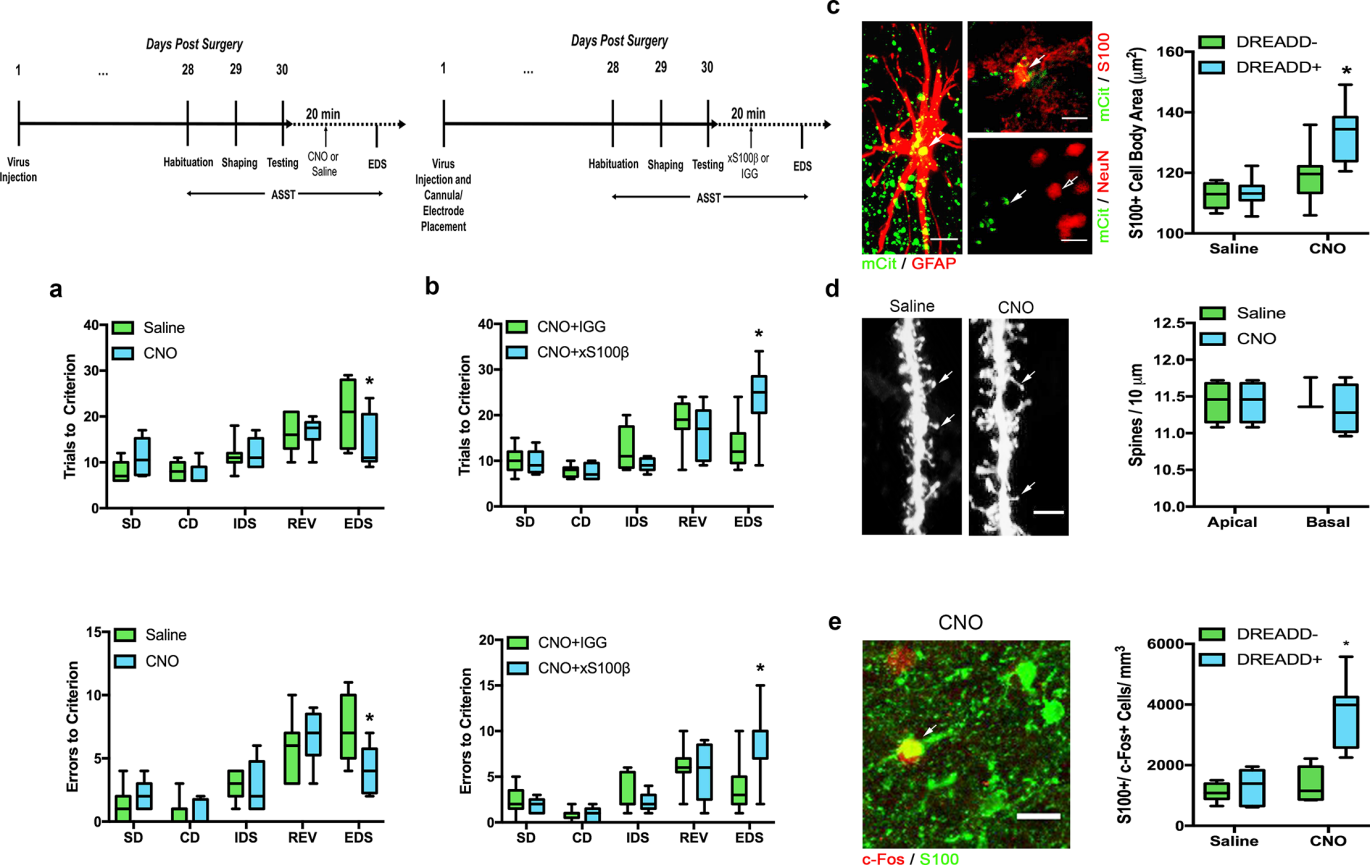
DREADD activation of astrocytes enhances cognitive flexibility. Activation of the GFAP-Gq-DREADD with CNO (n = 8) 20 minutes before completion of the extradimensional shift (EDS) enhanced cognitive flexibility relative to saline-treated controls (n = 7). A timeline for the GFAP-Gq-DREADD experiment is above. ***a*.** CNO-treated rats took fewer trials to reach criterion and made fewer errors on the EDS than saline-treated rats. ***b*.** In a separate cohort, CNO-treated rats that received infusions of antibody against S100β (xS100β) in the mPFC (n = 9) took more trials to reach criterion and made more errors on the EDS than CNO-treated rats that received infusions of IGG (n = 9). ***c*.** Confocal image showing Gq DREADD expression co-localized with GFAP+ astrocytes in the mPFC. Scale bar = 5 μm. Top: Co-localization of the astrocyte protein S100+ with Gq DREADD expression. Scale bar = 5 μm. Bottom: no co-localization between NeuN+ neurons (open arrow) and the Gq DREADD (closed arrow) was observed. Scale bar = 10 μm. S100+ astrocytes near mCitrine expression and in animals receiving CNO were larger compared to saline controls and when compared within-subjects to brain sections not containing mCitrine. ***d*.** Confocal images of DiI labeling in saline-treated and CNO-treated rats. Scale bar = 5 μm. In a separate cohort of GFAP-Gq-DREADD rats, CNO treatment (n = 4) did not alter dendritic spine density on either apical or basal dendrites of layer 2/3 pyramidal neurons in the mPFC relative to saline (n = 3). Arrows point to example spines along the labeled dendrites. ***e*.** Confocal image of a CNO activated S100+ astrocyte in green colocalized with the immediate early gene marker c-fos in red. Scale bar = 20 μm. Only S100+ astrocytes in close proximity to Gq DREADD expression in CNO-treated animals showed a significant increase in the density of colocalized S100+/ c-Fos+ cells. * p < 0.05 compared to controls. Arrows point to overlap in staining between c-Fos in red and S100 in green.

**Fig 6:**
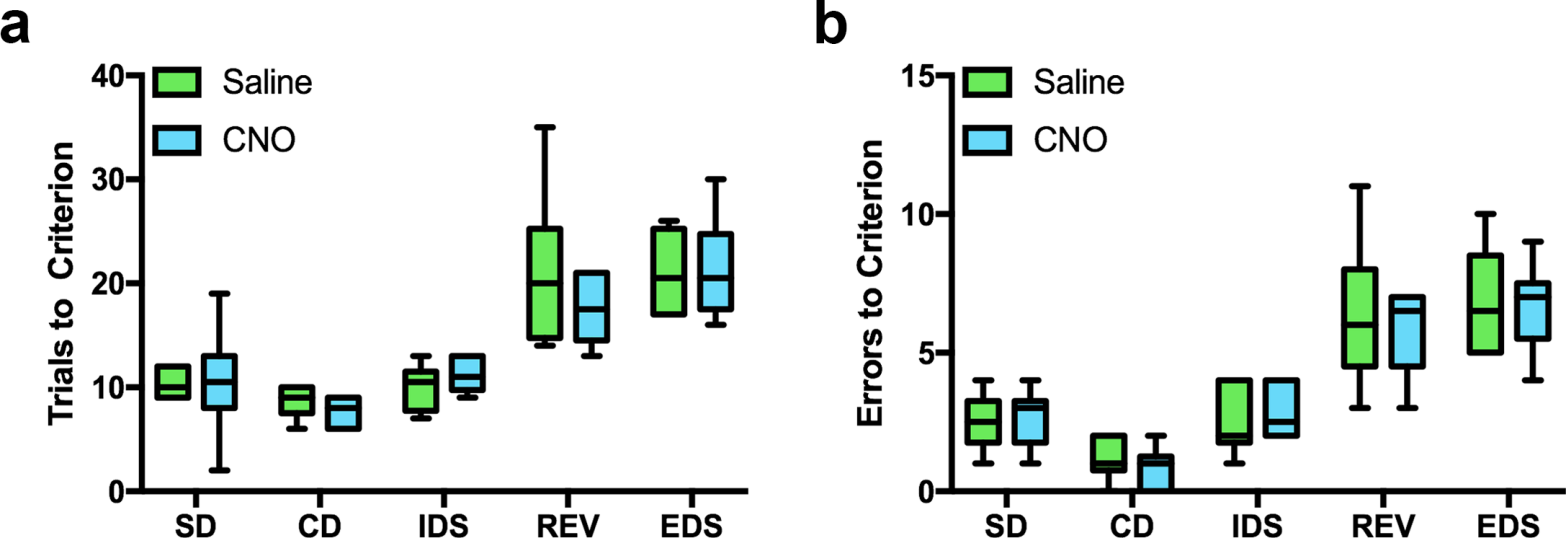
CNO treatment alone does not alter cognitive flexibility. Despite being thought of as an inert synthetic ligand, recent evidence suggests that CNO can activate endogenous receptors and have behaviorally relevant effects. To rule out the possibility that CNO treatment itself affects performance on the ASST, non-infected control rats (n = 6) were injected with CNO or saline 20 min prior to the completion of the extradimensional shift (EDS). ***a*,*b*.** There were no differences in number of trials to reach criterion or number of errors made between CNO- and saline-treated rats.

We investigated whether stimulating astrocyte Ca^2+^ signaling would improve cognitive flexibility. We verified that GFAP-Gq-DREADD expression was exclusively localized to astrocytes by demonstrating the presence of mCitrine in GFAP+ or S100+ cells, but not in NeuN+ cells (Fig 5C). We verified astrocyte activation by colocalizing S100+ and c-Fos labeled cells. There were significant main effects for condition (*F*_(1,13)_ = 16.87, *p* = .0012), expression (*F*_(1,13)_ = 66.19, *p* < .0001) and an interaction between condition and expression (*F*_(1,13)_ = 55.14, *p* < .0001) (Fig 5E). Bonferroni corrected post-hoc analysis revealed that astrocytes in CNO treated animals that were in close proximity to mCitrine expression had a greater density of colocalized S100+/ c-Fos+ cells (*p* < .0001) suggesting that CNO effectively activated astrocytes in the mPFC (Fig 5E). Activation of Ca^2+^ signaling in GFAP-Gq-DREADD-expressing astrocytes reduced the number of trials (*p* = .0133) and errors (*p* = .0117) to criterion on the extradimensional shift (Fig 5A). We also controlled for the possibility that CNO treatment alone, independent of GFAP-Gq-DREADD expression could alter performance on the extradimensional shift portion of the ASST. In a separate experiment, we administered CNO to non-infected control rats 20 minutes prior to the completion of the extradimensional shift. CNO did not alter performance on this portion of the ASST (Fig 6A and 6B). We next investigated whether CNO treatment in GFAP-Gq-DREADD-expressing astrocytes affected astrocyte morphology, similar to previous research associating larger astrocytes with improved cognition [23,29]. Activation of astrocytic Ca^2+^ signaling by CNO increased the size of transduced and neighboring astrocytes (Fig 5C) compared to control groups in astrocytes expressing the GFAP-Gq-DREADD. There were significant main effects for treatment (*F*_(1,26)_ = 23.28, *p* < .0001) and expression (*F*_(1,26)_ = 7.262, *p* = .0104), as well as an interaction effect (*F*_(1,26)_ = 5.717, *p* = .0243), suggesting that increases in astrocyte cell body size were driven specifically by activation of Ca^2+^ signaling in astrocytes (Fig 5C).

We also examined whether DREADD activation with CNO induced changes in dendritic architecture. GFAP-Gq-DREADD activation did not alter spine density measurements of layer 2/3 pyramidal neurons for both apical (*t*_(5)_ = .8760, *p* = .4211) and basal (*t*_(5)_ = .7600, *p* = .4815) dendrites (Fig 5D). No differences in spine length, spine head or spine type were detected (Tables 1–3).

**Table 1:** Assessment of changes in dendritic spine density following CNO administration. To rule out the possibility that enhancement of cognitive flexibility in GFAP-Gq-DREADD activated rats was due to changes in dendritic spines. No differences in the percentage distribution of spine types were detected between saline- (n = 3) and CNO-treated (n = 4) rats.

SPINE DENSITY	SPINE TYPE	CONDITION	MEAN ± SEM	statistics	*P*-value
***APICAL***					
	Filopodia	Saline:	22 ± 6.43	*t*_(5)_ = 1.153	*p* = .3011
		CNO:	15 ± 2.38		
	Thin	Saline:	42 ± 3.06	*t*_(5)_ = .5926	*p* = .5792
		CNO:	45.5 ± 4.5		
	Mushroom	Saline:	9.33 ± 3.53	*t*_(5)_ = .9413	*p* = .3898
		CNO:	15 ± 4.435		
	Stubby	Saline:	26.7 ± 4.06	*t*_(5)_ = .3652	*p* = .3652
		CNO:	24.5 ± 4.11		
***BASAL***					
	Filopodia	Saline:	19.3 ± 8.82	*t*_(5)_ = .7396	*p* = .4928
		CNO:	12 ± 5.60		
	Thin	Saline:	37.3 ± 7.06	*t*_(5)_ = 1.066	*p* = .3353
		CNO:	15 ± 2.38		
	Mushroom	Saline:	16.7 ± 5.81	*t*_(5)_ = 1.345	*p* = .2365
		CNO:	10 ± .817		
	Stubby	Saline:	26.7 ± 2.40	*t*_(5)_ = .9825	*p* = .3710
		CNO:	30 ± 2.31		

**Table 2:** Assessment of changes in spine head diameter following CNO administration. No differences in spine head diameter were detected between saline- (n = 3) and CNO-treated (n = 4) rats.

SPINE HEAD		mean ± sem	statistics	*P*-value
***APICAL***	Saline:	7462 ± .050	*t*_(5)_ = .2895	*p* = .7838
	CNO:	7249 ± .0512		
***BASAL***	Saline:	.8829 ± .105	*t*_(5)_ = 1.026	*p* = .3521
	CNO:	7891 ± .021		

**Table 3:** Assessment of changes in spine length following CNO administration. No differences in spine length were detected between saline- (n = 3) and CNO-treated (n = 4) rats.

SPINE LENGTH		mean ± sem	statistics	*P*-value
***APICAL***	Saline:	1.71 ± .063	*t*_(5)_ = .3517	*p* = .7394
	CNO:	1.74 ± .051		
***BASAL***	Saline:	1.764 ± .056	*t*_(5)_ = .1447	*p* = .8906
	CNO:	1.78 ± .081		

S100β is an astrocyte-specific Ca^2+^ binding protein that can be released by astrocytes and has been associated with enhanced rhythmogenic firing in neurons through the regulation of extracellular Ca^2+^ [16,25]. Because attention and attentional shifting, components of performance on the ASST, are associated with changes in neuronal oscillations specifically in the theta and gamma frequencies [29–31], we predicted that changes in S100β levels may underlie changes in neuronal signaling that reflect enhancement in cognitive flexibility. In order to determine whether GFAP-Gq-DREADD-induced enhancement of cognitive flexibility was influenced by S100β, we infused S100β antibody or a non-immune rabbit IGG into GFAP-Gq-DREADD-expressing rats treated with CNO. Compared to non-immune IGG controls, S100β antibody infusion 20 minutes prior to the extradimensional shift significantly increased the number of trials needed to reach criterion (*p* = .0001) (Fig 5B) and the number of errors made (*p* = .0001) (Fig 5B), suggesting that S100β levels may directly influence cognitive flexibility.

### Infusion of S100β improves cognitive flexibility and coupling between theta and gamma oscillations

**Fig 7:**
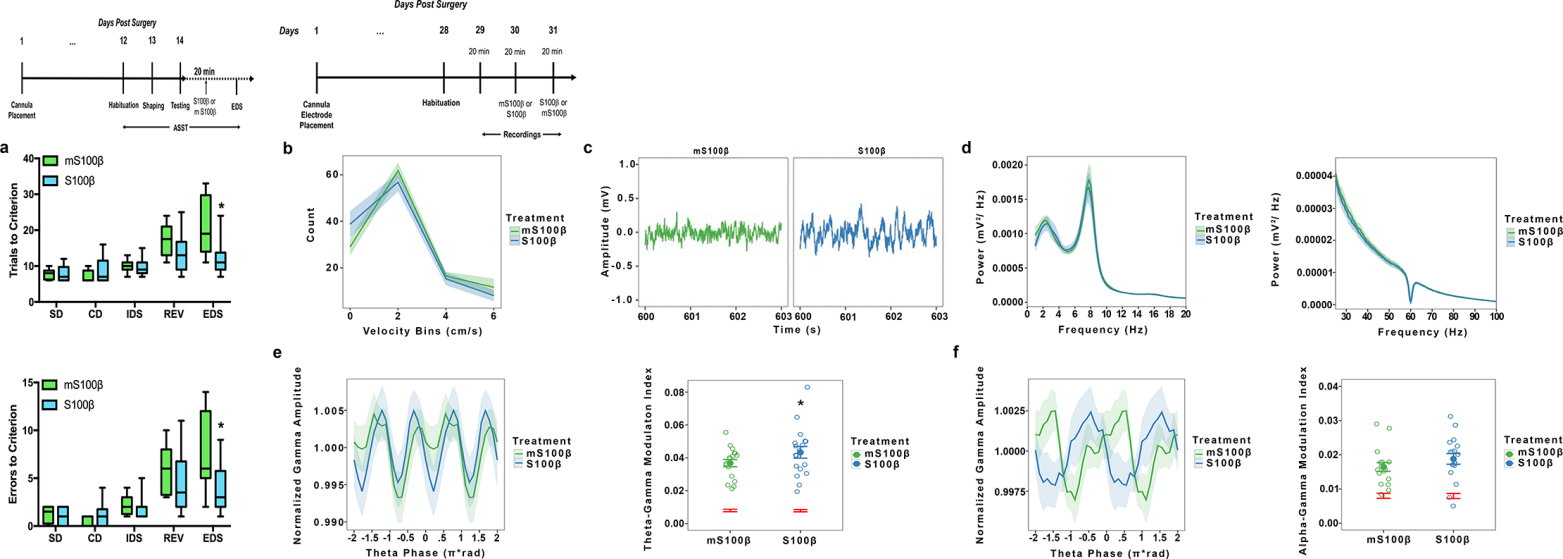
Infusion of the astrocyte-specific Ca^2+^ binding protein S100β in the mPFC enhances cognitive flexibility and theta-gamma coupling. To examine whether S100β facilitates cognitive function, rats were infused with either S100β (n = 12) or mS100β (n = 12), a mutated, nonfunctional form of S100β, in the mPFC 20 minutes before completing the extradimensional shift (EDS). To examine whether S100β infusion alters neuronal oscillations, a separate cohort of rats was infused with S100β followed by mS100β or vice versa (n = 18) in the mPFC and LFPs were recorded. A phase-amplitude coupling analysis was performed using within subject design to look at the role S100β plays in modulating the relationship between theta and gamma oscillations. Timelines for each experiment are provided above. For the behavioral experiment, the ASST occurred over 3 days, with the EDS performed on the last day. ***a*.** Rats infused with S100β required significantly fewer trials to reach criterion and made significantly fewer errors on the EDS compared to those infused with mS100β. ***b*.** A mixed effects model revealed no differences in movement across velocity bins. ***c*.** Raw LFP recordings from saline treated and L-AAA animals. ***d*.** Power spectra for low frequency ranges show no differences in the theta range. Power spectra for low frequency ranges show no differences in the gamma range. ***e*.** Phase amplitude coupling analysis plotting normalized gamma power as a function of theta phase. Permutation tests showed significant modulation above chance for both treatment conditions (red) with S100β significantly increasing theta-gamma modulation relative to mS100β controls, indicating that increasing S100β enhances theta-gamma coupling in the mPFC. * p < 0.05 compared to mS100β-infused. ***f*.** Phase amplitude coupling analysis plotting normalized gamma power as a function of alpha phase. Permutation results showed significant modulation about chance for both treatment conditions (red). S100β did not significantly increase alpha-gamma modulation relative to mS100β controls suggesting that S100β effects on neuronal oscillations may be specific to theta-gamma coupling in the mPFC.

We infused either S100β or mS100β into the mPFC of rats 20-minutes before completing the extradimensional shift. Infusion of S100β was associated with significantly fewer trials to reach criterion (*p* = .0001) and significantly fewer errors (*p* = .0001) (Fig 7A).

Using a within subject design, we treated a separate cohort of behaviorally naïve rats with mS100β and then S100β, or vice versa, and recorded LFPs from the over two 20 minute recordings sessions. We found no effect of treatment on locomotion (*t*_(17)_ = .9880, *p* = .3370) (Fig 7B). Infusion of S100β into the mPFC did not alter theta (*t*_(17)_ = .4690, *p* = .6450) or gamma (*t*_(17)_ = .8570, *p* = .4040) power individually (Fig 7C and 7D). However, we found that coupling between theta and gamma oscillations was significantly higher in animals treated withS100β compared to mS100β controls (*z*_(17)_ = 2.070, *p* = .0380) (Fig 7E). We also examined the modulation of gamma amplitude by alpha phase (12–20 Hz) and found no differences in coupling across groups (*z*_(17)_ = -1.336, *p* = .1820) (Fig 7F). These data suggest that astrocytes participate in cognitive flexibility in the mPFC by modulating the relationship between oscillations commonly associated with cognitive ability, namely theta and gamma, via S100β.

## Discussion

Here we show that reduction of astrocyte number in the mPFC impairs performance on the extradimensional shift of the ASST and diminishes power across several frequency ranges associated with cognitive flexibility. Conversely, GFAP-Gq-DREADD activation of astrocytes improves task performance, which can be prevented by blocking the activity of S100β. Finally, we show that S100β infusion into the mPFC is sufficient to improve cognitive flexibility and enhance synchrony between theta and gamma oscillations. Collectively, our results suggest that astrocytes in the mPFC play an important role in cognitive flexibility. While our results raise many questions, they also provide some of the first preliminary evidence suggesting that astrocytes participate in the complex signaling that underlies behavior.

It is important to note that astrocytes exhibit great morphological and biochemical heterogeneity [32,33]. Therefore, it is possible that different subpopulations of astrocytes serve different functions [32,33]. While some of the approaches we used, namely L-AAA, likely affected all subtypes of astrocytes equally, others targeted specific subpopulations of astrocytes, (i.e., GFAP or S100β), and this can make it difficult to draw mechanistic conclusions. Future research must develop tools for the study of specific subpopulations of astrocytes in rats in order to fully develop models for how astrocytes are specifically contributing to brain functioning. One of the few tools that enables specific targeting of astrocytes in rats is the use of viruses. Our GFAP-Gq-DREADD experiment achieved astrocyte-specific expression through the use of a GFAP promoter. GFAP-Gq-DREADD expression in approximately 10–15% of this subpopulation of astrocytes along with CNO treatment was sufficient to enhance cognitive flexibility associated with the mPFC. One concern is that the GFAP-Gq-DREADD and the S100β manipulations may be providing evidence about separate populations of astrocytes. These concerns are somewhat allayed by immunohistochemical data suggesting that 40–50% of S100+ cells co-express with GFAP [23], and electrophysiological data suggesting that activation of a single astrocyte often has excitatory effects on many neighboring astrocytes [14,15,17]. Nevertheless, we cannot rule out the possibility that both populations have unique functional outputs and future research should determine to what extent specific astrocyte subpopulations are functionally distinct.

Another potential limitation is the incidental damage to astrocytes caused by surgery [32–34]. Abnormalities in astrocyte density were evident in the controls of some of our experiments. In the L-AAA recording study, implanted animals in both vehicle and treatment groups had more astrocytes around the implant compared to non-operated controls, likely due to reactive gliosis, which is typical of chronic cannula/ electrode implantation [32–34]. Likewise, in the two experiments requiring cannula implants, similar increases in astrocyte number were observed. However, it is important to note that vehicle-treated cannulated animals performed the ASST like non-operated controls indicating that the presence of more astrocytes alone, at least in the mPFC, does not necessarily alter behavior.

The recent finding that CNO does not cross the blood brain barrier and is instead converted to clozapine [35] further complicates interpretation of some of our findings. While it is impossible to completely rule out the non-specific effects of systemic CNO administration, we did control for CNO by conducting separate experiments in non-GFAP-Gq-DREADD expressing animals. CNO treatment alone did not alter cognitive flexibility, and when combined with the data from the antibody against S100β experiment, it seems unlikely that there was significant interference of CNO alone, at least at the behavioral level.

While care needs to be taken in the interpretation of our findings, our results do expand on classic lesioning studies showing that a large reduction in the majority of neurons in the mPFC impairs performance on the extradimensional shift component of the ASST [22]. Our results suggest that a comparably small (approximately 20%) decrease in the number of astrocytes in the mPFC, in the absence of any substantial decrease in the number of neurons, induces an impairment on the extradimensional shift task that is essentially identical to that which has been observed with much larger scale neuron loss. We observed no differences in GFAP+ staining in brain regions in close proximity to the injection site. Our observations are consistent with previous work showing that differences in the number of GFAP+ cells were localized to the lesion site and not surrounding areas at the same concentration and injection volume of L-AAA used in our study [19]. Moreover, when a higher concentration of L-AAA was used to lesion astrocytes in the mPFC, astrocyte loss again was only localized to the targeted area, suggesting that at a relatively small injection volume and low concentration, L-AAA can be readily used to target astrocytes in individual brain regions [18]. Nevertheless, given astrocytes susceptibility to damage, it is possible that subtler differences in astrocyte populations, not detectable by immunohistochemistry, may result from L-AAA treatment. The use of L-AAA in the mPFC also resulted in a slight enhancement of performance on the compound discrimination, a task not thought to be dependent on mPFC functioning. One possible explanation for this paradoxical finding is that the mPFC provides inhibitory control over basic learning that when impaired, relinquishes its control and allows simpler forms of learning to occur more rapidly. These findings hint that mPFC influence might extend to more basic learning mechanisms as well.

Our results provide some evidence for a role for astrocytes in the regulation of cognitive flexibility, however, the exact mechanism(s) through which astrocytes contribute to behavior remains unclear. There is increasing evidence that astrocytic involvement in the regulation of metabolism and brain energy levels may be vital for the support of numerous brain functions [36,37]. In the rat hippocampus, learning has shown to lead to significant increases in extracellular lactate levels in astrocytes. Moreover, disruption of astrocytic release of lactate has been shown to induce amnesia and impair LTP, suggesting that astrocyte-neuron lactate transport is required for long-term memory formation and memory processing [38,39]. While we did not explore brain metabolism specifically, our L-AAA findings and GFAP-Gq-DREADD findings are largely consistent with hypotheses that astrocytes contribute to behavior, and more generally brain function, through the regulation of brain energy levels. In the case of L-AAA, the reduction of astrocytes likely impairs many astrocyte-specific functions, including the release of S100β, as well as metabolic functions such as the astrocyte-neuron lactate transport, which could combine to have negative effects on cognitive flexibility. However, our reduction of astrocyte number did not result in complete loss of mPFC function, and secondary measures of neuronal viability suggest that neurons in the mPFC were structurally and functionally unchanged. This suggests that perhaps the degree of behavioral impairment in the wake of astrocyte depletion may exist on a continuum, where modest loss of astrocyte function impairs specific behaviors/ function, while greater loss, as seen in patients with mood disorders and schizophrenia, may result in more global impairment. Moreover, our results using the GFAP-Gq-DREADD showed that viral-mediated activation of astrocytic signaling in the mPFC improved cognitive flexibility. While our results suggest a specific role of S100β in mediating this improvement, given previous research showing that activation enhances astrocytic metabolic functions [38,39], we cannot exclude the possibility that this improvement may in be due, in part to a boost in several astrocytic signaling modalities including those devoted to the maintenance of brain energy levels. Whether S100β plays a role in astrocytic regulation of brain metabolism is relatively understudied, and future research should investigate this possibility. Nonetheless, our findings, in concert with the evidence from the role of astrocytes in brain metabolism, support the idea that astrocytes are vital contributors to brain function, and that abnormalities in astrocytes can have significant negative consequences for brain function.

Deficits in astrocyte function have been shown to negatively impact neuronal oscillations, however an exact mechanism by which astrocytes contribute to rhythmic firing is less clear [15]. In our experiments, both L-AAA and S100β infusion altered neuronal oscillations across frequency ranges commonly thought to support cognitive flexibility, but which are also impaired in many patients with mood disorders or schizophrenia [3]. Moreover, our data support previous findings suggesting that S100β plays a crucial role in the regulation of rhythmic firing in neurons [16,25]. The idea that astrocytes are important for the support of brain rhythms is consistent with evidence showing that brain-wide disruption of astrocytes impairs gamma rhythms [15] and recent evidence examining the role of the astrocytic gap junction subunit connexin-43 [40]. Cre-dependent knockout of the connexin-43 gene in astrocytes in the brains of mice resulted in an abnormal sleep-wake cycle, presumably due to astrocyte-induced silencing of orexin neurons in the lateral hypothalamic area [40]. While examining the role of connexin-43 was beyond the scope of our study, these findings raise the possibility that in our L-AAA treated rats, one consequence of the use of a general astrocyte toxin may be the reduction of connexin-43 which could contribute to the abnormalities in rhythmic firing observed. In the case of S100β it is currently unknown whether S100β release or trafficking is altered by connexin-43, but future research should explore this possibility.

The effect sizes of our LFP findings, particularly the S100β-induced enhancement of coupling between theta and gamma oscillation are small, which makes understanding the specific mechanism underlying cognitive flexibility difficult. We feel that this difference, while small, is still an important finding in that studies exploring astrocyte functioning in traditionally high order brain regions are lacking. However, while promising and consistent with previously published data [3, 15, 16, 25], we recognize that more research needs to be done in order to confirm and solidify this potentially understudied role of astrocytes. Similarly, it is also important to note that we recorded LFPs in awake behaving rats but not engaging in a task of cognitive flexibility, and therefore caution needs to be taken in interpreting our results. This limitation makes it difficult to say whether our behavioral findings resulted from the direct consequence of decreased power across gamma, alpha, and delta frequency ranges in the case of the L-AAA lesion study and improved coupling between gamma and theta oscillations in the case of the S100β infusion study, or some other task-dependent change in neuronal firing that we were unable to observe. While we recognize this as a shortcoming in our approach, the data still suggest that astrocyte related manipulations have effects on neuronal oscillations, and that these changes likely contribute, in some way, to differences in behavioral performance.

### Conclusions

While it has long been recognized that astrocytes support neurons by serving a wide array of functions from maintenance of the blood brain barrier and metabolic function [36,37,41] to the regulation of extracellular glutamate [42,43] and the clearance of toxins during sleep [44], few studies have linked this support to cognitive functions specifically. Increasing evidence suggests that abnormalities in astrocyte number and physiology may be directly [18,19] or indirectly [44] responsible for impaired functioning associated with neuropsychiatric disorders [8]. Our results suggest that a loss of astrocytes in the mPFC diminishes cognitive flexibility and neuronal oscillations associated with task performance, while augmentation of astrocyte signaling in the mPFC enhances cognitive flexibility possibly through the astrocytic release of S100β. Collectively, these results raise many questions but also suggest a novel role for astrocytes in the mPFC in the regulation of cognitive flexibility.
